# DNA Content of Human Tumours: Change in Uterine Tumours During Radiotherapy and their Response to Treatment

**DOI:** 10.1038/bjc.1959.87

**Published:** 1959-12

**Authors:** B. M. Richards, N. B. Atkin


### DNA CONTENT OF HUMAN TUMOURS: CHANGE IN UTERINE TUMOURS DURING RADIOTHERAPY AND THEIR RESPONSE TO TREATMENT

B. M. RICHARDS* AND N. B. ATKIN

*Wheatstone Physics Laboratory, King's College, Strand, London, W.C.2 and Department of Cancer Research, Mount Vernon Hospital and Radium Institute, Northwood, Middlesex*

Received for publication October 31, 1959

IONIZING radiations are widely used in the treatment of cancer in man and frequently they produce excellent results. Sometimes, however, cases are encountered which give very poor results although they are of a type that can be expected to respond well to radiotherapy. Although these cases are in the minority (a few per cent of the total) they remind us that response to radiotherapy can vary considerably between different tumours in any one histopathological group. An additional complication is that the immediate response, viewed clinically or histologically, does not necessarily reflect the ultimate success or failure of the treatment. These problems, both practical and biological, have been clearly annotated by Merrill (1958) in a review of the various attempts that have already been made to classify radiation response in terms of " radiosensitivity " and " radiocurability ". A great need still exists, therefore, for an *objective* method for predicting radiosensitivity and radiocurability ; the search for such a method is impeded in no small degree by the lack of understanding of the effects of radiation on the fundamental processes of dividing cells.

For several years we have been measuring the DNA contents of human tumours both before treatment (which has formed the basis of a previous report (Atkin and Richards, 1956)) and in some cases during radiotherapy and, if possible, at later stages such as at operation. Most of our data refer to uterine tumours partly because they are common and partly because the type of radiotherapy which they receive frequently makes it possible to obtain biopsies at several intervals during the course of treatment. We have now collected sufficient results to warrant some account of the types of effect of the radiation treatment on the pattern of DNA values of the tumours and to consider these results with a view to obtaining a quantitative cytochemical method for assessing radiocurability.

In addition to their practical interest, these observations are of value in the study of the effects of irradiation on a dividing cell population. An extensive literature on this subject already exists but it contains few instances where the effects on the fundamental chemical processes, such as DNA synthesis, have been examined at the single cell level. Our contribution is at a considerable disadvantage compared with an experimental study because we have no " controls ", and the factors of the irradiation treatment are those dictated by the clinician. In particular we must accept the fact that the dose received at any point in the tumour is difficult to assess and standardize. Nevertheless, as we have previously suggested

* Now at Medical Research Council Biophysics Research Unit, King's College, London, W.C.2.

(Atkin and Richards, 1956), the information that can be obtained from human tumours may be of more direct relevance to the study of this disease in man than perhaps much that is obtainable from studying transplantable tumours of animals. The relative advantages of investigating spontaneous and transplantable tumours have recently been considered by Scott (1958).

### MATERIALS AND METHODS

The details of our methods of preparation of tissues and measurements of Feulgen stain have been described previously (Atkin and Richards, 1956). One improvement that has been made since that report, is that the tumour material is now fixed by methanol freeze substitution at the hospital, thus avoiding the delay incurred in transport to King's College before fixation. Feulgen staining is also done at the hospital and the Feulgen-stained specimens are transported mounted in non-drying immersion oil, after having been dehydrated in the alcohol series and passed through xylene. If necessary the stained preparations are stored for short periods at 2° C. Immediately before measurement specimens are rehydrated and mounted in glycerol. This is necessary because the cell-crushing procedure, which is part of the measuring technique, does not work satisfactorily with immersion oil as a mountant. The amounts of Feulgen stain per cell nucleus were measured with the scanning photometer (Deeley, 1955).

The results for the DNA content (amount of Feulgen stain) for 60–100 tumour cells in each tumour are plotted as frequency histograms. In some cases the scale of the amount of DNA is logarithmic instead of linear because some specimens, particularly after irradiation, show ranges of DNA values over several multiples of ploidy. The method that we previously used for calibrating the amounts of stain, so that different specimens may be compared, was also used here : in this a sample of 10–30 inflammatory cells, usually polymorphonuclear leucocytes, is measured in every specimen ; these act as a standard which we designate the '*l*' value and the scale of amounts of DNA in the histograms is thus in terms of *l*.

### RESULTS

The limitations of any system of classification of human tumours are emphasized by the differences in response of the tumours in any one group to the same method of radiation treatment. This variation in response between individual cases may appear in the early stages of treatment or only after completion of treatment. Likewise, in the results reported here, the effect of irradiation on the DNA content and pattern of DNA values in tumours may vary between individual tumours in both early and late stages of treatment.

The results described here are limited to those on tumours of the uterus for the reasons given in the Introduction. In a parallel paper (Atkin, Richards and Ross (1959)) we have considered our results, both those presented here and from other tumours, in relation to the system of clinical staging that has been standardized for some years, and also to other factors, e.g., age of patient. In this report we have attempted to discuss the changes produced by the radiation treatment in the pattern of DNA values in all the cases in which we have been fortunate enough to have obtained specimens before, during and occasionally after the course of treatment. Table I includes the relevant data on pathology and treatment for the cases which we have selected for illustration in this paper.

TABLE I.—*Table of Data for Cases Described*

| Case No. | Age | Site | Stage | Pathology | Treatment | Subsequent history | Fig. No. |
|---|---|---|---|---|---|---|---|
| 501 | 48 | Cervix | I | Poorly differentiated squamous cell carcinoma (P.d.sq.c.ca.) | 2 Stk.* + Wert.* | Died from widespread metastases 4 months later | 1 |
| 491 | 51 | Corpus | — | Moderately well differentiated columnar cell adenocarcinoma | 1 Stk. + total hysterectomy | Well 8 months later | 2 |
| 161 | 73 | ,, | I | Moderately well differentiated columnar cell papillary adenocarcinoma | Intracav.$Co^{60} \times 2$ | Well 41 months later | 3 |
| 421 | 43 | Cervix | I | Moderately well differentiated keratinizing squamous cell carcinoma (exophytic) | 3 Stk. | Well 19 months later | 4 |
| 449 | 58 | ,, (stump) | II | P.d.sq.c.ca. | Ra. ovoids (60 mg. × 30 hr.) Wert. | Well 9 months later | 5 |
| 486 | 33 | Cervix | I | P.d.sq.c.ca. with slight keratinization | 3 Stk. | Well 21 months later | 6 |
| 280 | 51 | ,, | II | Keratinizing sq.c.ca. | Ra. (modified Paris technique) + Wert. | Recurrence left side of pelvis 12 months later | 7 |
| 72 | 59 | ,, | II | P.d. adenoacanthoma | 3 Stk. + DXR to parametria | 2 months later, colostomy (mass in pelvis and local recurrence). Died a few weeks later | 8 |
| 537 | 47 | ,, | II | P.d. adenoacanthoma | 3 Stk. | 2 months later at laparotomy : inoperable tumour invading rectum : course of DXR to pelvis. Subsequently developed local recurrence in cervix and died 8 months after first treatment | 9 |

* Stk. = Stockholm radium insertion.

* Wert. = Wertheim's hysterectomy.

The cases have been classified into groups according to the type of change shown in the pattern of DNA values. Out of a total of 29 cases where specimens were obtained during treatment, 6 showed only very slight evidence of change shortly after the first radiation treatment (3–7 days), 20 cases showed an effect that for various reasons may be regarded as a typical response to irradiation, and the remaining 3 cases, although they gave the typical response at first, had several features which suggested that they were unusual. It is interesting that 2 of the 3 unusual cases were similar in certain important respects. In the following account we shall describe examples of each of the above categories.

FIG. 1.—Carcinoma of the cervix : before treatment ; 7 days after first radium insertion ; at operation 4 weeks after first treatment.

1. *Slight change*

Although the conditions of irradiation of the tumours differed somewhat in detail according to the individual needs of the case the differences do not seem to be sufficient to explain the fact that 6 out of 29 cases show little, if any, early change in their pattern of DNA values, in contrast to the marked changes seen in the remaining cases.

An example of this type of case is given in Fig. 1 (case No. 501) which is a Stage I cervical carcinoma. The basic DNA value (i.e., the post-telophase DNA value of most of the tumour cells which is usually indicated by a prominent mode near the lower limit of the histogram) is seen to be approximately the same for all three tumour samples. Although there are slight indications of change at day 7, such as a reduction in the height of the primary mode and increase in the frequency of higher DNA values, the pattern at day 28 is that of a typical dividing cell population. This impression is strengthened by the fact that all stages of mitosis were observed in parallel aceto-orcein preparations. It is noteworthy, however, that this case received only 2 Stockholm doses, the treatment being completed by operation.

Fig. 2 and 3 show two cases of carcinoma of the uterine body (case No. 491 and 161) which were treated by radium and by cobalt ($Co^{60}$) insertions (Strickland,

FIG. 2.—Carcinoma of the corpus: before treatment; 3 days after radium insertion.

FIG. 3.—Carcinoma of the corpus: before treatment; 7 days after first treatment with intracavitary $Co^{60}$.

1953) respectively. In neither case can any significant change be detected at the short times after irradiation at which second specimens were available but in both these cases the specimen may have been taken too soon after the beginning of irradiation for marked changes to have occurred.

Although all 6 cases, of which the aforementioned 3 are examples, failed to show significant changes in tumour samples taken during treatment, the clinical response in each case was good, except in case No. 501 where the presence of mitoses in the Wertheim specimen indicated that active tumour remained, but since 4 of these cases (including No. 501) were operated upon (total hysterectomy or Wertheim's hysterectomy) at varying times following radiotherapy, the success of the radiation treatment alone could not be assessed.

2. *Typical changes*

Apart from the 6 cases mentioned in the previous section, all cases studied showed significant changes in the early stages of treatment (i.e. at 7 days). These

FIG. 4.—Carcinoma of the cervix : before treatment ; 7 days after first radium insertion.

FIG. 5.—Carcinoma of the cervix (stump) : before treatment ; 14 days after first radium insertion.

changes are regarded as typical of the early effects of irradiation on the pattern of DNA value because they appear in most tumours irrespective of additional or subsequent changes (or of the final outcome of the treatment). These changes are to some extent similar to those that have been observed as radiation effects on DNA content in the cells of transplantable animal tumours (see later).

Case No. 421 (Fig. 4), an exophytic Stage I cervical carcinoma, shows a striking accumulation of cells with larger DNA values, and, in this case, the histogram has definite modes at exact multiples of the basic DNA value. This is a characteristic pattern which is frequently seen at 7 days following the first radiation treatment. These higher values are also commonly found at 14 days after a radium insertion, as for example, in Fig. 5 (case No. 449, a Stage II cervical tumour). The appearance of higher multiples of DNA values is to be expected from the well-known "giant cell" formation after irradiation seen in histological studies.

Both cases described so far in this section were near-diploid tumours, but the typical radiation response of increasing frequency of higher ploidy multiples is also found with near-tetraploid (2 *l*) tumours. An example of this is case No. 486 (Fig. 6), a cervical carcinoma, which at day 7 after the first radium insertion shows a prominent mode at near-octoploid (4 *l*) and also values in excess of 16 *l*. Seven days later (after a second insertion of radium at day 7) higher multiple values persist but there is some suggestion of a mode at the original basic DNA value.

In all 3 cases so far described the last histogram represents the last occasion on which viable cells were present in samples taken during treatment ; subsequent samples, where available, contained no tumour, and 6 months follow-up examina-

FIG. 6.—Carcinoma of the cervix : before treatment ; 7 days after first radium insertion ; 7 days after second radium insertion.

tions gave favourable results. The typical changes seen in the early stages of radiation treatment probably reflect only the sensitivity of the majority of the tumour cells to radiation and do not imply that the tumour as a whole is curable, because such response changes are found in both successfully and unsuccessfully treated cases.

### 3. *Unusual changes*

Some of the cases that we have had the opportunity to examine have been noteworthy for one of two reasons. Firstly, they may have shown unusual features in the changes in their pattern of DNA values brought about by the radiation treatment, or secondly, they may have shown radioresistant properties which make them important from the clinical standpoint. Clearly, our aim is to see whether we find cases that are noteworthy for both reasons, or better still, if those that have a poor prognosis have patterns of DNA values *before treatment* which are unusual. In this way it might be possible to obtain an objective quantitative criterion of radioresistance. We shall now describe some of the cases in which we have encountered unusual results and later attempt to assess the value of our observations with respect to such a criterion.

An interesting case was one treated at the Middlesex Hospital by a modified Paris radium technique in which it was possible to obtain a biopsy very much sooner after the beginning of treatment than the customary 7 days (for the Stockholm method). This was a Stage II cervical tumour, the results for which

FIG. 7.—Carcinoma of the cervix : before treatment ; 24 hours after commencement of treatment ; 5 days after commencement of treatment ; at completion of radiation treatment ; at operation 31 days after commencement of treatment (radium was inserted for two periods of 3 days).

are illustrated in Fig. 7 (case No. 280). Radium insertions were given for two periods : day 0 to day 3, and day 5 to day 8. It is noteworthy that the change in the pattern of DNA values towards the appearance of higher multiple values is not found at 1 day after the beginning of irradiation, whereas when 5 days have elapsed, during three of which the tumour was exposed to radiation, there is a very marked change in this direction. The patient underwent a Wertheim's hysterectomy at day 31 when the small residuum of tissue found indicated that the tumour was radiosensitive. After 4 months, however, a recurrence appeared in the left side of the pelvis.

As already mentioned we have found two cases in which the radiation treatment seems to have had a more interesting effect on the tumour than the typical one of increasing the frequency of cells with higher DNA content ; the typical effects in the early stages are nevertheless present. The unusual feature is the fact that the pattern of DNA content in the tumour after the end of radiotherapy differs from that present in the untreated tumour in a manner not found in other cases. The first of these is given in Fig. 8 (case No. 72—a Stage II cervical carcinoma).

FIG. 8.—Carcinoma of the cervix (adenoacanthoma) : before treatment ; 7 days after first radium insertion ; 14 days after second radium insertion ; biopsy specimens of actively-growing tumour at primary site obtained on days 51, 70 and 73.

In this case, the pre-treatment biopsy is itself unusual with a relatively flat distribution ; the basic DNA value is difficult to determine owing to the lack of a definite mode but is probably very near the *l* value. The tumour failed to regress after radium treatment by a modified Stockholm technique so that tumour material was available at day 7 and 21 during radium treatment, at day 51 and 70 after treatment, and finally at day 73 when laparotomy was performed. Soon after the last operation the patient died.

The samples at day 7 and day 21 both show the typical increase in the frequency of higher DNA values of which, however, relatively few exceed 4 *l* ; cell divisions were noted in each sample, and anaphases and telophases were seen at day 7 (i.e., very soon after irradiation). Almost 2 months after the beginning of treatment there was actively growing tumour at the primary site ; this shows a fairly definite mode of DNA values at about $1\frac{1}{2}$ $l$. In both subsequent tumour samples this mode is very pronounced, and there is a range of values going up to but rarely exceeding 3 $l$. This pattern of DNA values differs very much from that of the original tumour and hence it appears that a new actively growing tumour strain had emerged. The radiation treatment may have selected a resistant cell lineage having a different DNA content from the majority of the original tumour cells.

Many of the unusual features of this case (No. 72) were also found in another (case No. 537—Stage II cervical carcinoma), the DNA values for which are given in Fig. 9. Unfortunately, biopsies were available at day 0, 7 and 81 only. In the pre-treatment sample (day 0) an extremely wide range of DNA values is found,

FIG. 9.—Carcinoma of the cervix (adenoacanthoma) : before treatment ; 7 days after first radium insertion ; biopsy specimen of local recurrence on day 81.

i.e. well outside the twofold range in which most of the cells of a dividing cell population usually lie. Like the previous case the pre-treatment distribution is essentially flat, but here the total range of values is much wider than twofold. Although values near $l$ are frequent, it is not possible accurately to assign a figure for the basic DNA value. Seven days after the first radium insertion the characteristic increase in the frequency of higher values has occurred but at day 81, when laparotomy was performed, there is evidence of a mode of values appearing between $l$ and 2 $l$. There are some indications, therefore, that the changes in the pattern of DNA values following treatment are similar to those found in the case last described, while a comparison of the clinical features and prognosis of the two cases reveals many similarities. Shortly after the end of radiation treatment a considerable amount of tumour remained. In this case (No. 537) the tumour remained inoperable and, moreover, proved resistant to a further course of deep X-rays. It is interesting that both these tumours which had been dramatically unresponsive to radiation treatment and which showed a pattern of DNA values unlike that seen for other tumours, should have been identified as " mixed " tumours according to the histological classification of Glücksmann and Cherry (1956). These authors also find such tumours to be frequently radioresistant.

### DISCUSSION

Radiotherapeutic techniques have achieved a considerable measure of success in the treatment of many types of human tumours. It has long been known that ionizing radiations have a greater immediate effect on reproducing cells than on differentiated cells. In this respect, however, radiation probably affects tumour and normal dividing cells in the same manner. The success of the techniques, therefore, has depended largely on the ability of the radiotherapist to design the exposure to radiation in a way that the dose received by adjacent normal tissue is minimal and the maximum dose is delivered to the malignant cells.

Despite the substantial research effort which in recent years has been directed to the study of radiation effects both immediate and long-term (see, for example, Gray, 1956; Scott, 1958; Errera, 1959), comparatively little is known about the effects of ionizing radiation on cells in terms of disruption of the vital chemical processes. One of these vital processes is the synthesis of DNA. This has been shown by many authors (Swift, 1950; Howard and Pelc, 1951; Walker and Yates, 1952; Richards, Walker and Deeley, 1956) to occur during the interphase of the mitotic cycle, and X-ray doses of about 1250–2000 r affect the rate of this synthesis. As judged from isotope studies on animal tumours, regenerating liver and normal tissues, there is a reduction of the rate which, after a single exposure, may last for up to about 40 hours (Howard, 1956; Holmes, 1947; Kelly *et al.*, 1957; Nygaard and Potter, 1959). This effect is temporary, therefore, and the cells can recover, complete their DNA synthesis and pass through a normal mitosis. Complete cessation of DNA synthesis usually requires very much higher doses than 2000 r.

Very small doses of radiation can cause mitotic delay. The results of Caspersson, Klein and Ringertz (1958) have shown very clearly that doses of 1250 r on certain animal ascites tumours while not preventing DNA synthesis, although there may be a temporary cut in the rate, cause an accumulation of cells at the pre-mitotic DNA content. After recovery from irradiation these cells probably pass through a normal mitosis. It is noteworthy, however, that one of the typical effects which we noted in our results, namely, the "doubling-up" of pre-mitotic cells by abortive mitosis with nuclear reconstruction without cell cleavage, endomitosis or endoreduplication, did not appear to occur under the experimental conditions used by Caspersson *et al.* (1958). Twenty-three out of 29 cases of human tumours showed this "doubling-up" which probably had occurred more than once in those cells which later appeared with DNA contents 4 or 8 times higher than the basic DNA value of the tumour. This difference may be explained simply by the possibility that human tumour cells can undergo endomitosis more readily than the animal tumour cells, but the conditions of the irradiation (dose and dose rate, state of oxygenation, etc.) were certainly not the same for the animal tumours as for our clinical cases, so that the difference might be due to one or more of several possible factors. It is noteworthy, however, that the majority of the cases described here showed similar changes in the early stages of treatment despite the fact that the conditions of irradiation could not have been the same for all cases.

The most interesting of the cases which we described here were those (2 out of 29) which showed a high degree of radioresistance (i.e., lack of regression and absence of widespread necrosis) and were in fact incurable by radiation treatment; their clinical and pathological features were remarkably similar. The micro-spectrophotometric observations that we made suggest a possible reason for this radioresistance ; comparison of the pattern of DNA values in the pre-treatment biopsies with that in biopsies taken during or after treatment provide strong evidence that a selection of cells has occurred. Before treatment the histogram is flat, being without any definite mode and in one case (No. 537) the range of DNA values is sufficiently wide to accommodate much aneuploidy ; in addition, both cases show histological evidence that the tumours were composed of more than one type of cell. The slides of these tumours were kindly studied by Dr. Glücksmann who found them to be " mixed tumours " according to his criteria (Glücksmann and Cherry, 1956). After treatment a definite mode of DNA values appeared at an intermediate position between $l$ and $2\ l$, suggesting that the tumours now consisted mainly of one type of cell (with respect to DNA content) being the progeny of a strain of cells which might have been selected out of the original mixed population by virtue of a greater radioresistance than their neighbours. In the absence of competition and perhaps because they were able to use the products of necrosis of killed cells, a phenomenon that has been observed in experiments on animal tumours (Révész, 1955, 1958), this strain of cells gave rise to recurrent tumour.

The most urgent question arising from our results is whether or not they allow such radioresistant tumours to be distinguished from others before their method of treatment is decided. Certainly the pre-treatment pattern of DNA values is unusual in lacking a definite mode but this is not sufficient evidence, nor are our own results based on a sufficiently large number of cases at present, to recommend that a patient be treated other than by radiotherapy, if for clinical reasons this is considered to be the method of choice.

### SUMMARY

1. Measurements have been made of the DNA content of cells from 29 human uterine tumours before, during and after treatment by intracavitary radium or radio-cobalt.

2. Six cases showed only slight changes in the pattern of DNA values at 3–7 days after the first treatment. Twenty cases showed the " typical " effect of irradiation, as seen at 7 or 14 days after the first treatment : there was an accumulation of cells having large amounts of DNA, which usually were multiples of the basic DNA value (2, 4 or 8 times). The remaining cases presented certain unusual features.

3. The changes that have been observed have been briefly discussed in relation to the effect of ionizing radiations on DNA synthesis and the mitotic process.

4. While the " typical " response, with the appearance of cells having high DNA values, was seen in tumours which responded well to radiotherapy, 2 tumours which responded poorly were characterized by the appearance of a new modal DNA value in the triploid region. These 2 tumours, which histologically were adenoacanthomata, were also characterized by an unusual pattern of DNA values *before* treatment. The possibility that changes in the DNA content of cells during the early stages of radiotherapy, or perhaps the pattern of DNA values found before treatment, might form the basis of an objective test of radiosensitivity is discussed.

Thanks are due to the radiotherapeutic and gynaecological staff of Mount Vernon Hospital who provided material used in this study, and to Miss M. D. Snelling for kindly allowing material from a case treated at the Middlesex Hospital to be included.

Dr. H. B. Fell, F.R.S., and Dr. M. H. F. Wilkins, F.R.S., kindly read the manuscript, and helpful suggestions were made by Dr. L. H. Gray, Dr. Alma Howard and Mr. D. E. A. Jones. The technical assistance of Mr. D. Doxey is acknowledged. Expenses were defrayed by grants from the British Empire Cancer Campaign.

### REFERENCES

ATKIN, N. B. AND RICHARDS, B. M.—(1956) *Brit. J. Cancer*, **10**, 769.
*Iidem* AND ROSS, A. J.—(1959) *Ibid.*, **13**, 773.
CASPERSSON, T., KLEIN, EVA AND RINGERTZ, N.—(1958) *Cancer Res.*, **18**, 857.
DEELEY, E. M.—(1955) *J. sci. Instrum.*, **32**, 263.
ERRERA, M.—(1959) Chapt. 15 " Effects of Radiation on Cells " in ' The Cell ', Ed. Brachet and Mirsky, Vol. I. New York (Acad. Press).
GLÜCKSMANN, A. AND CHERRY, CORA P.—(1956) *Cancer*, **9**, 971.
GRAY, L. H.—(1956) *Ann. Rev. nuc. Sci.*, **6**, 353.
HOLMES, B. E.—(1947) *Brit. J. Radiol.*, **20**, 450.
HOWARD, ALMA.—(1956) " Influence of Radiation on DNA Metabolism ", in *Ciba Symp.* on Ionizing Radiations and Cell Metabolism. London (Churchill).
*Idem* AND PELC, S. R.—(1951) *Exp. Cell Res.*, **2**, 178.
KELLY, LOLA S., HIRSCH, J. D., BEACH, G. AND PETRAKIS, N. L.—(1957) *Proc. Soc. exp. Biol., N.Y.*, **94**, 83.
MERRILL, J. A.—(1958) Chapt. 7 " Cytohistological Evaluation of Radiation Response in Carcinoma of the Cervix : Its Present Clinical Significance ", in ' Progress in Radiation Therapy ', Ed. Buschke. New York (Grune and Stratton).
NYGAARD, O. F. AND POTTER, R. L.—(1959) *Radiation Res.*, **10**, 462.
RÉVÉSZ, L.—(1955) *J. nat. Cancer Inst.*, **15**, 1691.—(1958) *Ibid.*, **20**, 1157.
RICHARDS, B. M., WALKER, P. M. B. AND DEELEY, E. M.—(1956) *Ann N.Y. Acad. Sci.*, **63**, 831.
SCOTT, O. C. A.—(1958) *Advanc. biol. med. Phys.*, **6**, 121.
STRICKLAND, P.—(1953) *J. Obstet. Gynaec. Brit. Emp.*, **60**, 898.
SWIFT, H. H.—(1950) *Physiol. Zoöl*, **23**, 169.
WALKER, P. M. B. AND YATES, HELEN.—(1952) *Symp. Soc. exp. Biol.*, **6**, 265.


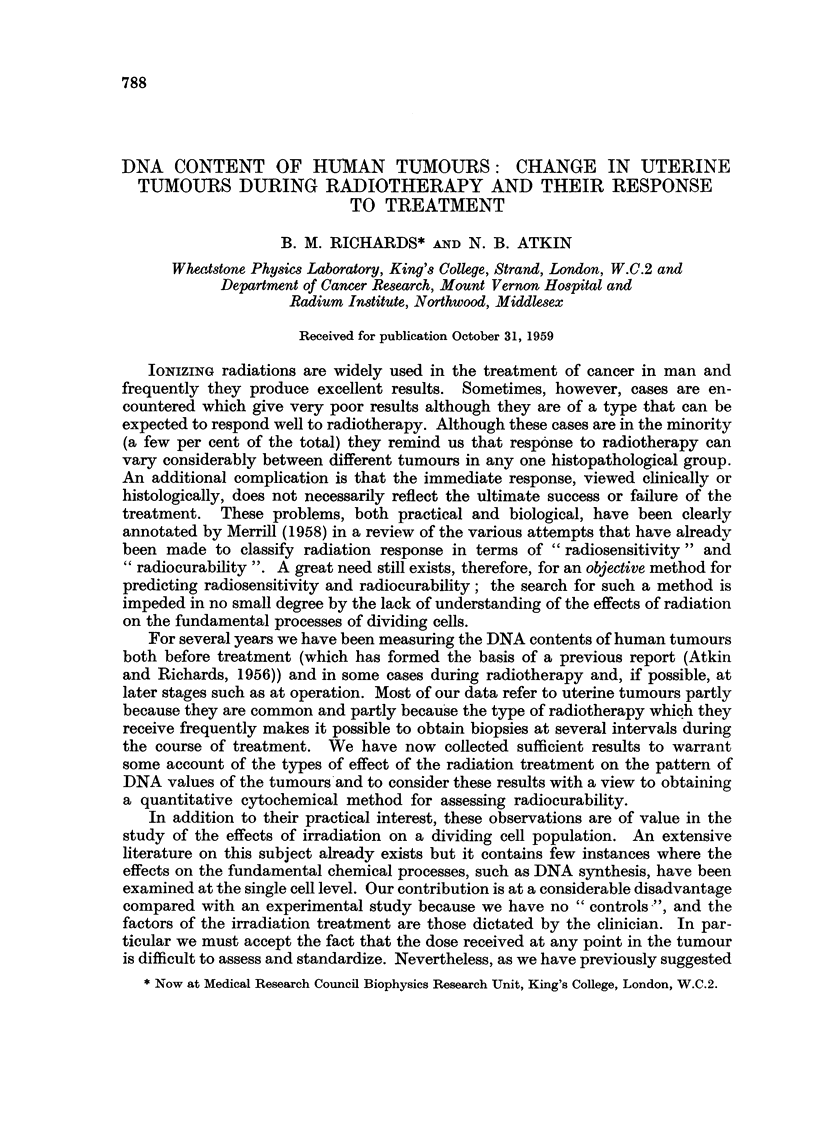

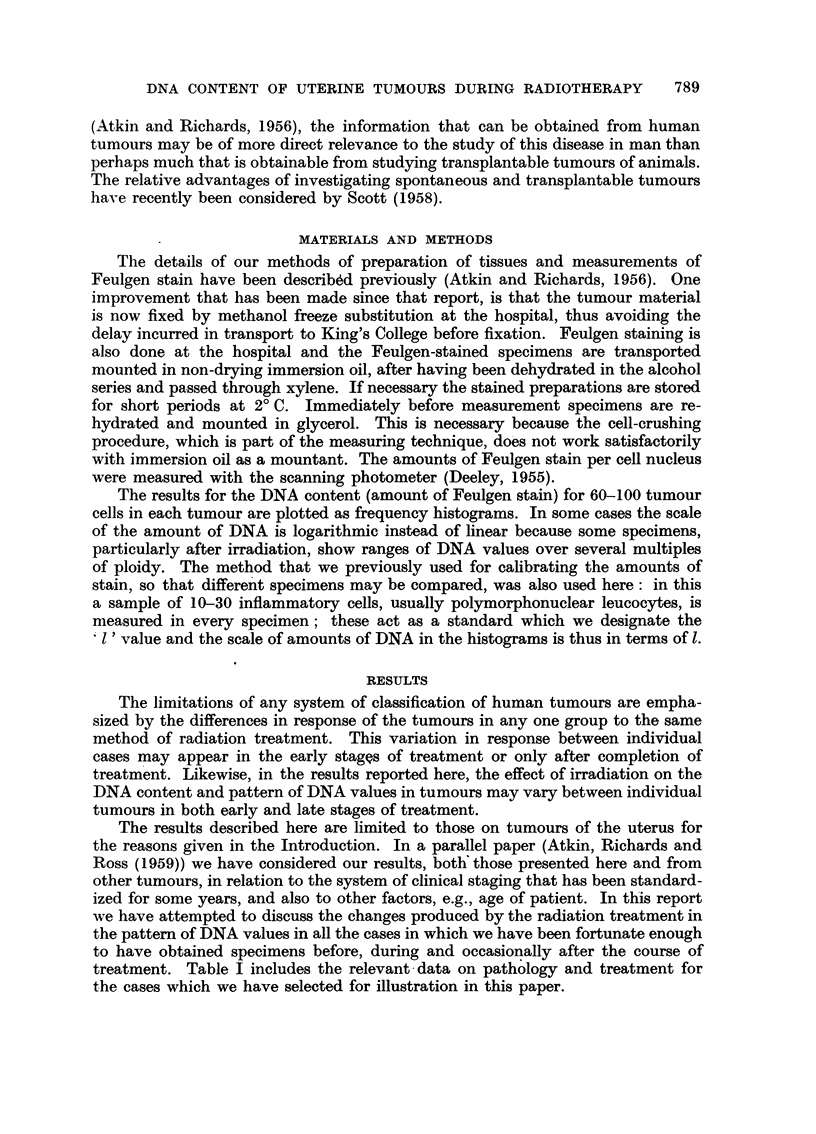

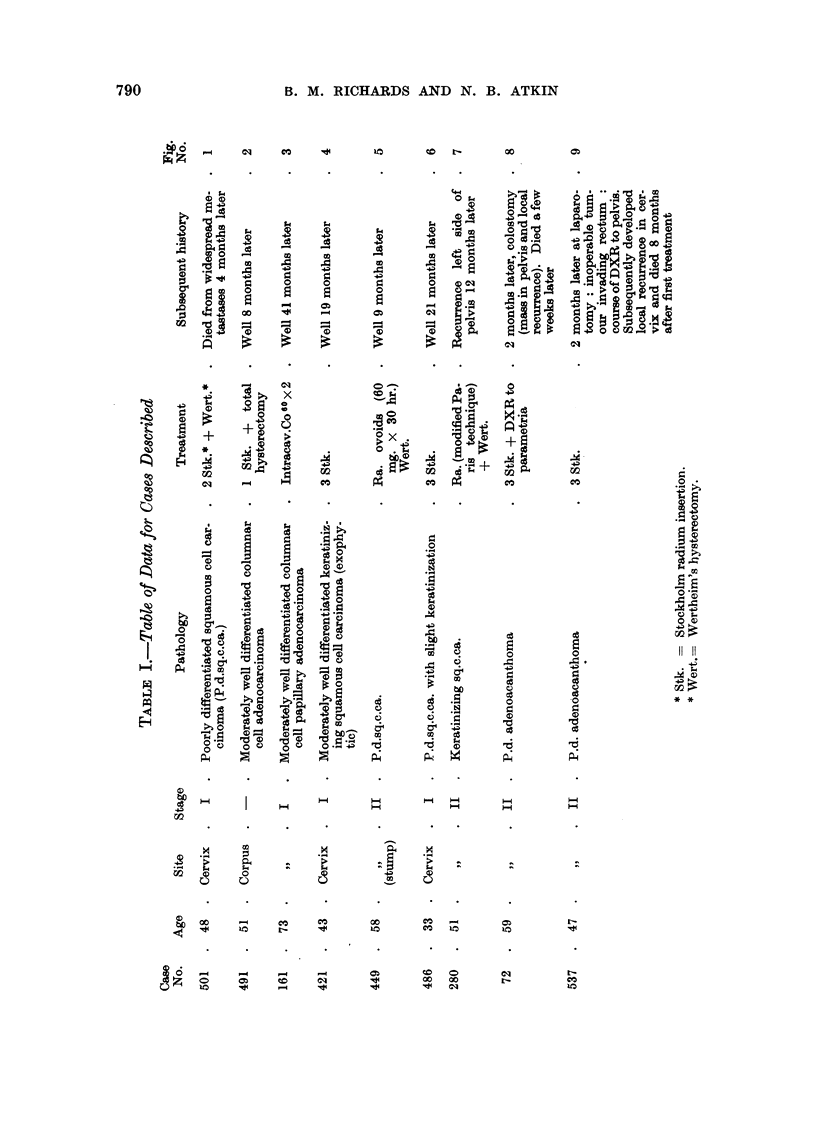

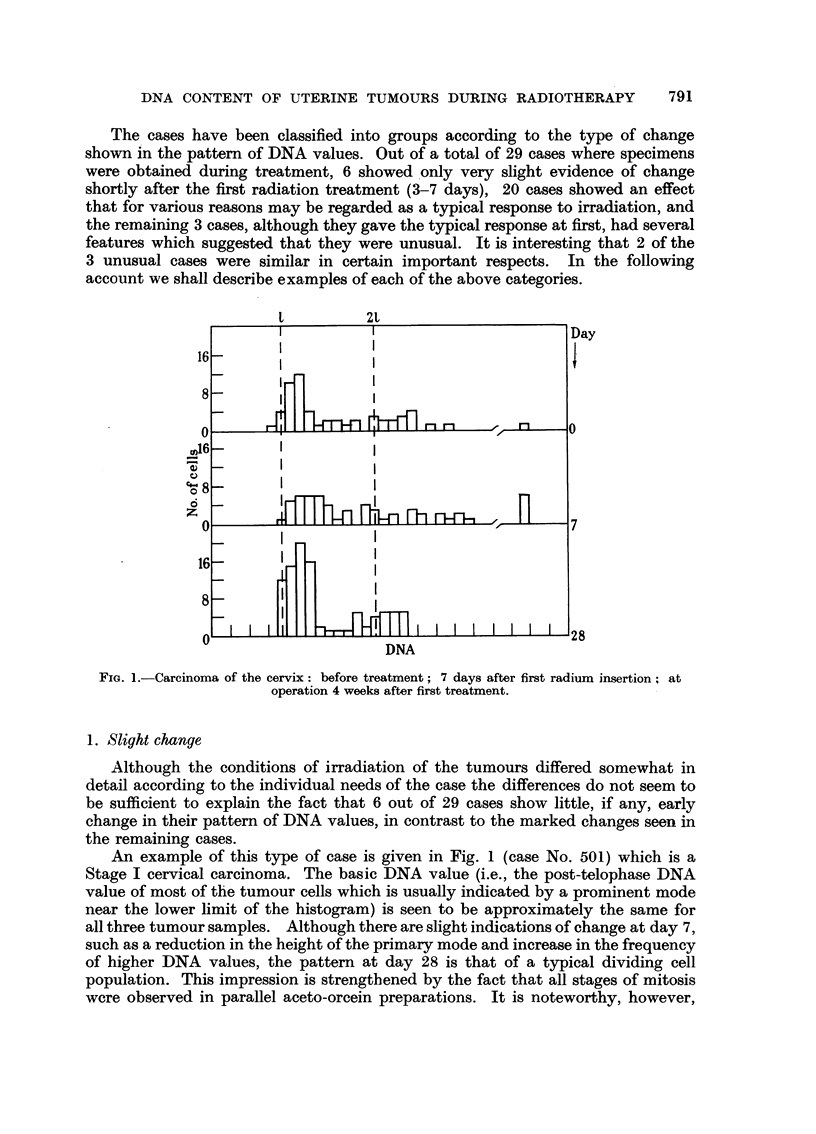

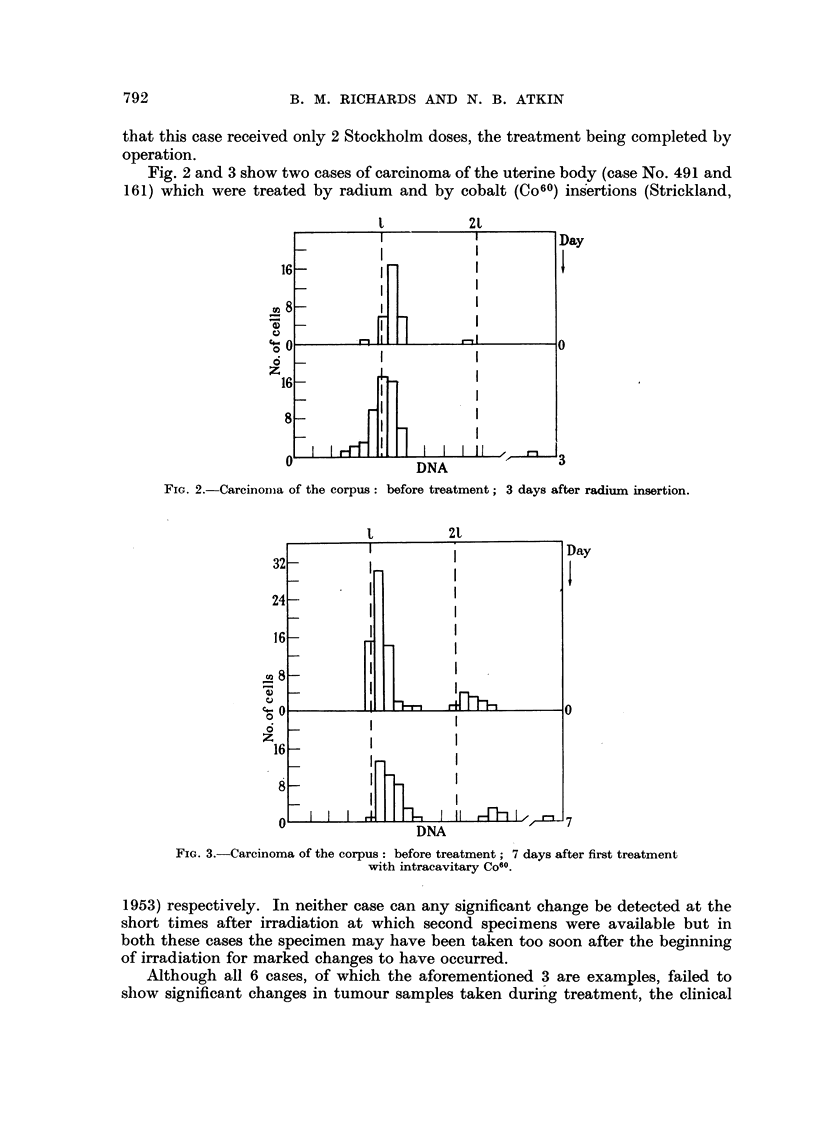

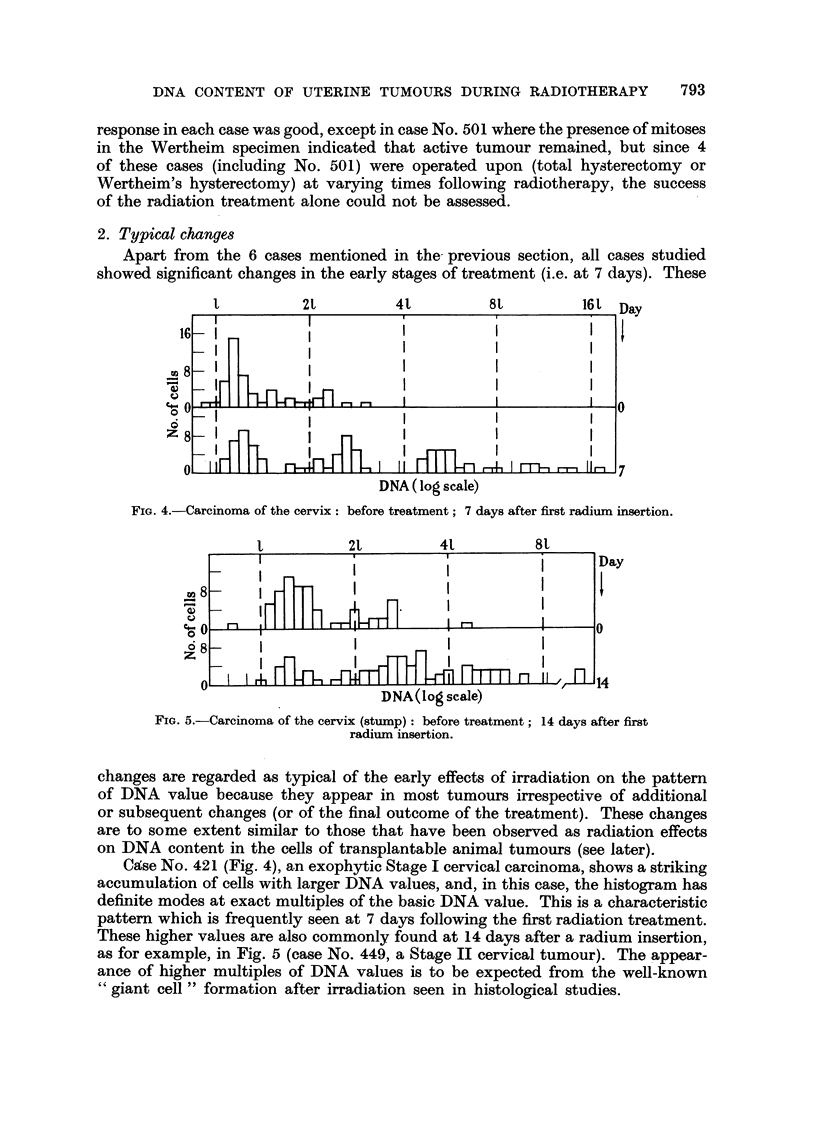

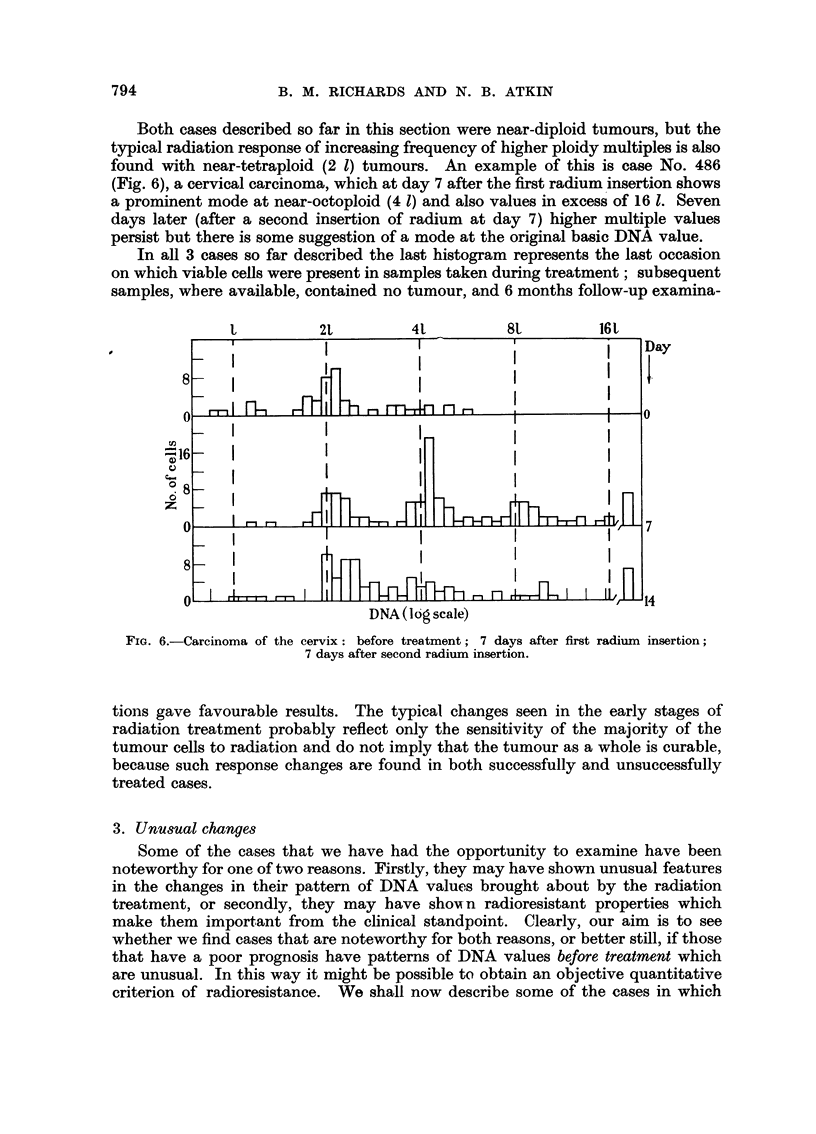

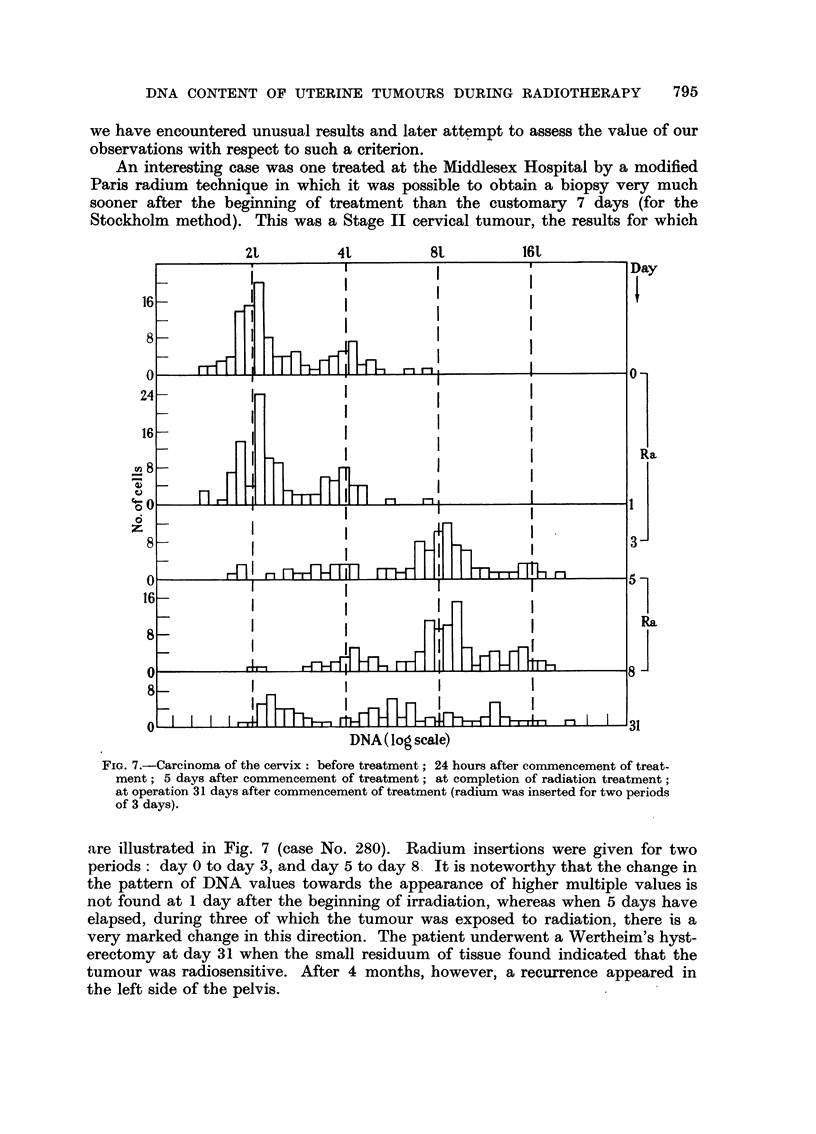

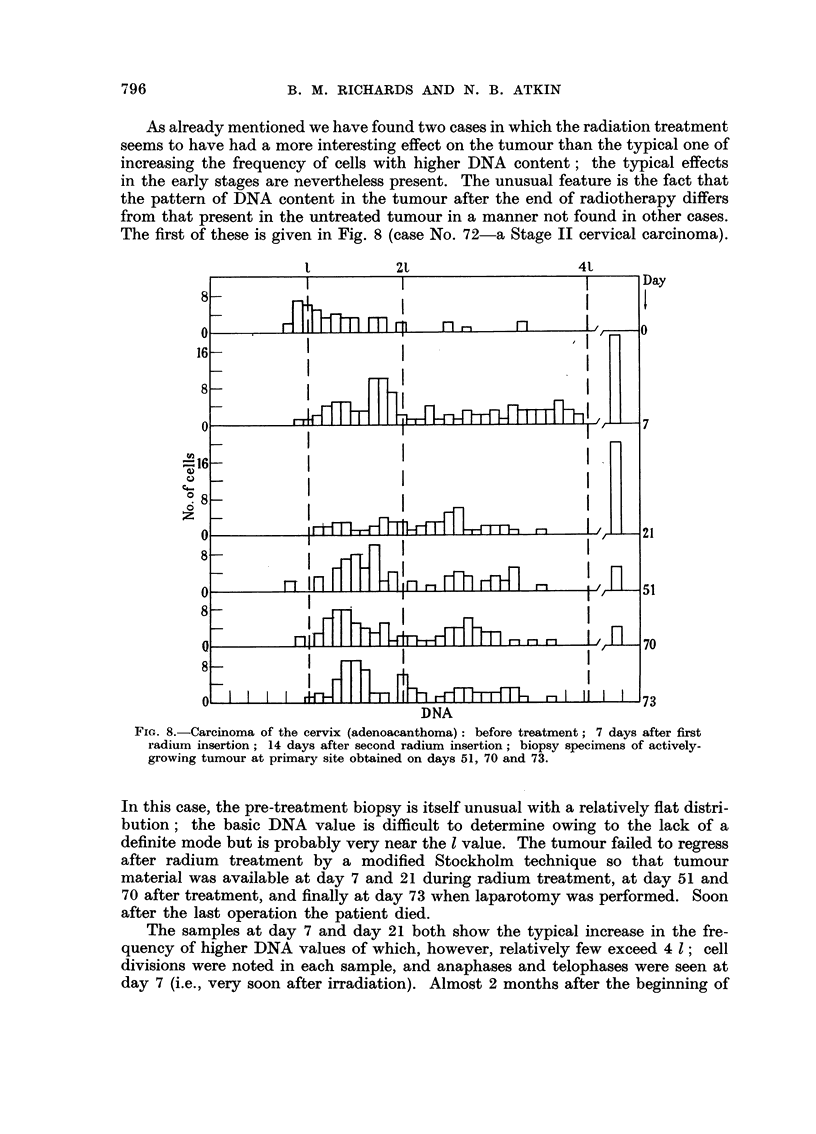

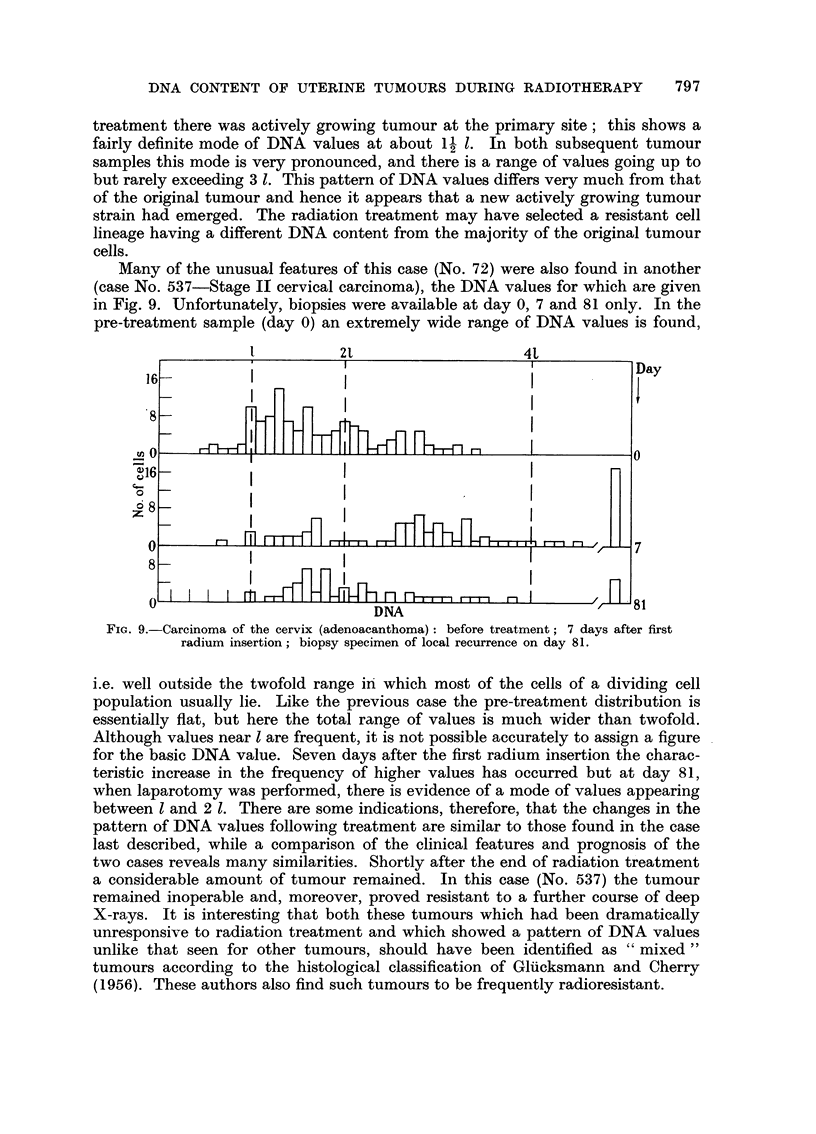

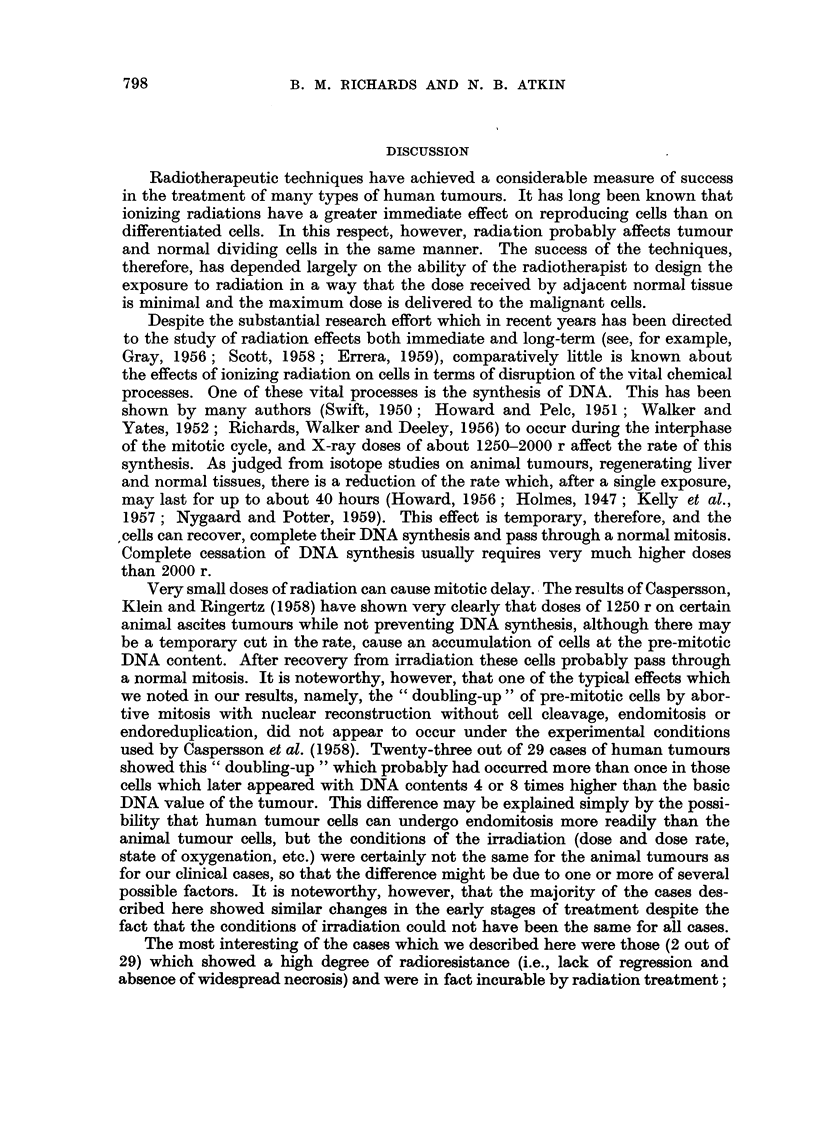

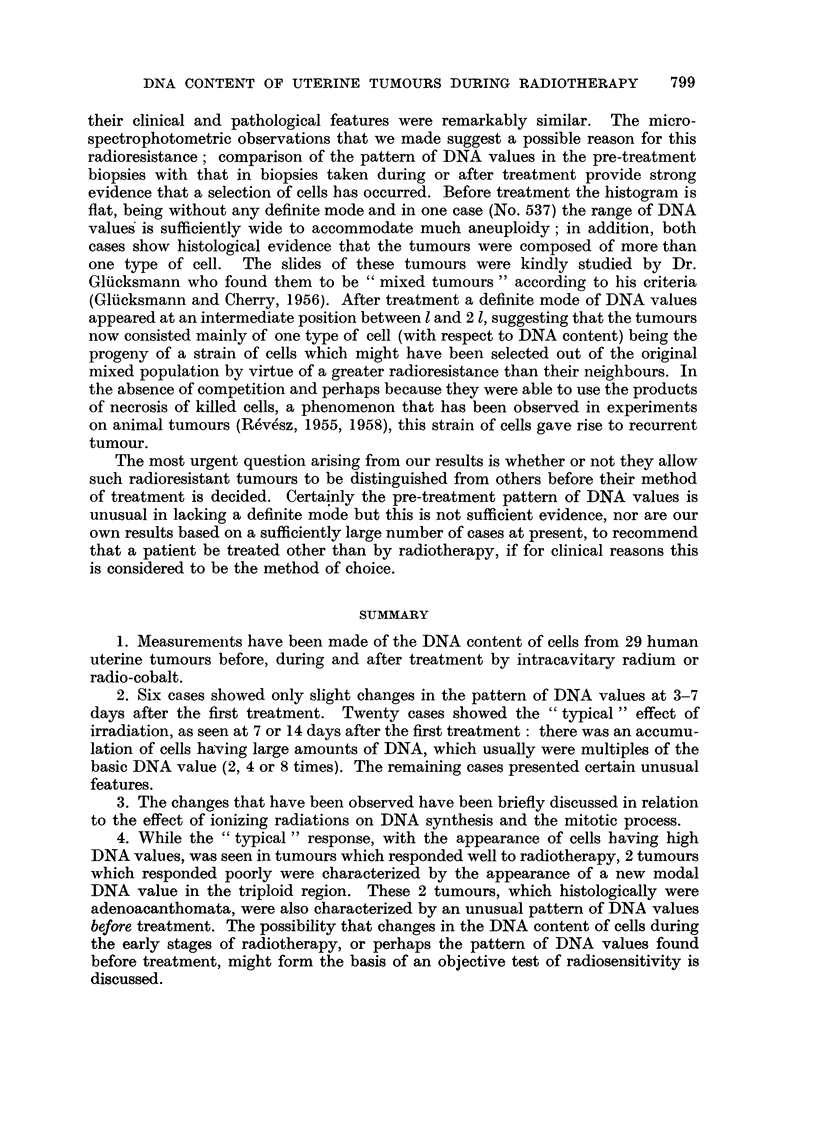

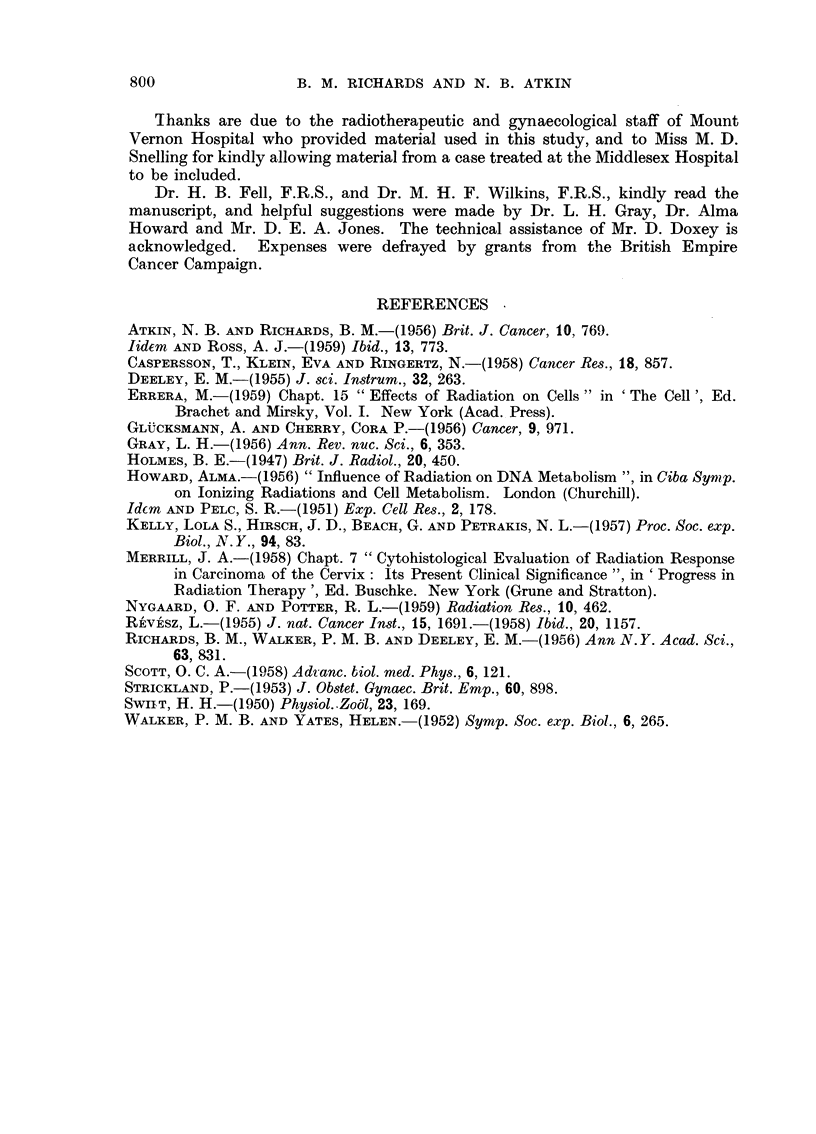


## References

[OCR_00711] ATKIN N. B., RICHARDS B. M., ROSS A. J. (1959). The deoxyribonucleic acid content of carcinoma of the uterus: an assessment of its possible significance in relation to histopathology and clinical course, based on data from 165 cases.. Br J Cancer.

[OCR_00714] CASPERSSON T., KLEIN E., RINGERTZ N. R. (1958). Cytochemical studies on some effects of x-radiation on three ascites tumors.. Cancer Res.

[OCR_00722] CHERRY C. P., GLUCKSMANN A. (1956). Incidence, histology, and response to radiation of mixed carcinomas (adenoacanthomas) of the uterine cervix.. Cancer.

[OCR_00738] NYGAARD O. F., POTTER R. L. (1959). Effect of x-radiation on DNA metabolism in various tissues of the rat. I. Incorporation of C14-thymidine into DNA during the first 24 hours postirradiation.. Radiat Res.

[OCR_00742] RICHARDS B. M., WALKER P. M., DEELEY E. M. (1956). Changes in nuclear DNA in normal and ascites tumor cells.. Ann N Y Acad Sci.

[OCR_00748] STRICKLAND P. (1953). Carcinoma of the uterine body treated with radioactive cobalt.. J Obstet Gynaecol Br Emp.

